# Quality of life after pulmonary embolism: validation of the French version of the PEmb-QoL questionnaire

**DOI:** 10.1186/s12955-014-0174-4

**Published:** 2014-12-03

**Authors:** Mathilde Rochat, Marie Méan, Andreas Limacher, Olivier Hugli, Frederikus A Klok, Danny M Cohn, Drahomir Aujesky

**Affiliations:** Department of Internal Medicine, Lausanne University Hospital, CHUV, Rue du Bugnon 46, 1011 Lausanne, Switzerland; Division of General Internal Medicine, Bern University Hospital, Bern, Switzerland; Department of Clinical Research, and Institute of Social and Preventive Medicine (ISPM), CTU Bern, University of Bern, Bern, Switzerland; Emergency Department, Lausanne University Hospital, Lausanne, Switzerland; Department of Thrombosis and Hemostasis, Leiden University Medical Center, Leiden, the Netherlands; Department of Vascular Medicine, Academic Medical Center, Amsterdam, the Netherlands

## Abstract

**Background:**

The PEmb-QoL is a validated 40-item questionnaire to quantify health-related quality of life in patients having experienced pulmonary embolism (PE). It covers six health dimensions: frequency of complaints, activities of daily living limitations, work-related problems, social limitations, intensity of complaints, and emotional complaints. Originally developed in Dutch and English, we sought to prospectively validate the psychometric properties of a French version of the PEmb-QoL.

**Methods:**

We performed a forward and backward translation of the English version of the PEmb-QoL into French. French-speaking consecutive adult patients with an acute, objectively confirmed PE admitted to the emergency department of a Swiss university hospital between 08/2009 and 09/2011 were recruited telephonically. We used standard psychometric tests and criteria to evaluate the acceptability, reliability, and validity of the French version of the PEmb-QoL. We also performed an exploratory factor analysis.

**Results:**

Overall, 102 patients were enrolled in the study. The French version of the PEmb-QoL showed good reliability (internal consistency, item–total and inter-item correlations), reproducibility (test-retest reliability), and validity (convergent, discriminant) in French-speaking patients with PE. The exploratory factor analysis suggested three underlying dimensions: limitations in daily activity (items 4b-m, 5a-d), symptoms (items 1a-h and 7), and emotional complaints (items 9a-f and j).

**Conclusion:**

We successfully validated the French version of the PEmb-QoL questionnaire in patients with PE. Our results show that the PEmb-QoL is a valuable tool for assessing health-related quality of life after PE in French-speaking patients.

**Electronic supplementary material:**

The online version of this article (doi:10.1186/s12955-014-0174-4) contains supplementary material, which is available to authorized users.

## Introduction

Acute venous thromboembolism (VTE), defined as deep vein thrombosis (DVT) and/or pulmonary embolism (PE), is common and has a high impact on morbidity, mortality, and costs of care [1,2]. Besides the transient discomfort related to acute VTE, health-related quality of life is substantially influenced by the development of VTE-related complications [3]. The long-term natural course in patients surviving an acute VTE event can be complicated by recurrent episodes of VTE, bleeding complications caused by anticoagulation treatment, the post-thrombotic syndrome and in rare cases, chronic thromboembolic pulmonary hypertension [4,5].

Disease-specific quality of life questionnaires are necessary to better detect treatment effects and change over time in patients having the same disease. While instruments to measure disease-specific quality of life exist for patients with DVT [6], the Pulmonary Embolism Quality of Life (PEmb-QoL) questionnaire was only recently developed to specifically address health-related quality of life in patients having experienced PE [7,8].

The PEmb-QoL, originally developed in Dutch and translated into English, is a 40-item questionnaire that measures the impact of PE on quality of life from the patient’s perspective over the past four weeks [7,8]. A study using the Dutch version of the PEmb-QoL found that patients with PE had an impaired quality of life compared to the age-matched general population [9]. Recently, a Norwegian version of the PEmb-QoL was successfully validated [10]. Given that the PEmb-QoL questionnaire is the only available validated instrument to assess QoL after PE [8], we aimed to prospectively validate the psychometric properties of a French version of the PEmb-QoL questionnaire (Additional file 1).

## Methods

### PEmb-QoL questionnaire

The PEmb-QoL questionnaire contains nine questions (40 items) covering six dimensions: frequency of complaints (Q1, 8 items), activities of daily living limitations (Q4, 13 items), work-related problems (Q5, 4 items), social limitations (Q6, 1 item), intensity of complaints (Q7 and Q8, 1 item each), and emotional complaints (Q9, 10 items). Responses are rated on a Likert response scale.

Because no French version of this questionnaire is available, we performed a forward-backward translation from the English version of the PEmb-QoL questionnaire into French according to previous published recommendations [11]. In a first step, two independent native French speakers, of whom one was a naïve translator without medical background, performed a forward translation from the original English version into French. In a second step, two naïve English speakers performed a backward translation into English. A committee of three experts reviewed all translations and reached a consensus on any discrepancy. The final French version is shown in the supplemental online appendix.

### Scoring the PEmb-QoL questionnaire

The scales of Q1, Q4, Q5, and Q9 were reversed, with a low point score indicating a better quality of life. Two questions (Q2 ‘At what time of day are your lung symptoms most intense?’ and Q3 ‘Compared to one year ago, how would you rate the condition of your lungs in general now?’) were not scored. Item 4a was considered missing if the answer was ‘I do not work’. As described in the initial publication [8], the PEmb-QoL dimension scores were calculated by taking the mean of the constituting items. Dimension scores were then transformed to a scale from 0–100 to make them comparable across dimensions, with higher scores indicating worse outcome. To estimate the overall impact of PE on quality of life, we developed a PEmb-QoL summary score. In a first step, we transformed all item scores to a scale ranging from 0 to 100. In a second step, we averaged these transformed scores (except items Q2 and Q3) to obtain an overall summary score.

### Study subjects

We identified consecutive patients aged >18 years with an acute, objectively confirmed PE admitted at the emergency department of the Lausanne university hospital, Switzerland, from August 1, 2009 to September 30, 2011 using the hospital’s electronic patient tracking system. The confirmation of PE was based on either a high-probability ventilation-perfusion lung scan or a positive computed tomography scan [12,13]. We telephonically invited all screened patients who survived the PE episode for study participation. Exclusion criteria were refusal to participate, insufficient spoken language ability in French, history of dementia based on chart review, and residence in a nursing home or outside Switzerland.

We chose a sample size of 100 patients to validate the French version of the PEmb-QoL questionnaire, which is in accordance with methodological recommendations [14,15] and a previous similar validation study [16]. The local ethics committee (Commission cantonale (VD) d’éthique de la recherche sur l’être humain) approved the study and all patients provided written consent.

### Data collection

Eligible, consenting patients received a baseline PEmb-QoL and a French language version of the Short-Form Health Survey (SF-36) questionnaire per mail [17]. The SF-36 questionnaire is a well validated generic quality of life measure consisting of 36 items grouped into eight dimensions (physical functioning, social functioning, physical role functioning, emotional role functioning, mental health, vitality, bodily pain, and general health). The scores vary from 0 to 100 for each dimension, with higher values indicating better health [18,19]. The SF-36 also provides a physical and mental health summary score. Standardized dimension and summary scores of the SF-36 questionnaire were calculated using the U.S. 1998 reference population [20].

Patients were asked to complete and return both questionnaires using a pre-stamped return envelope. Participants were then mailed a second PEmb-QoL and SF-36 questionnaire ten days after the baseline evaluation. In case a patient returned an incomplete questionnaire, a study collaborator contacted the patient by telephone to complete all missing items.

We used patient medical records to collect the following baseline characteristics for all enrolled patients: age, gender, cardiopulmonary comorbidity (defined as any cardiac disease with systolic or diastolic ventricular dysfunction or any obstructive or restrictive pulmonary disease), active cancer (defined as cancer with ongoing oncologic or palliative treatment within the previous six months), obesity (defined as body mass index more than 30 kg/m^2^), history of prior VTE, and the time interval between the index PE and study inclusion.

### Psychometric evaluation of the French version of the PEmb-QoL questionnaire

We used standard statistical tests and criteria to evaluate the acceptability, reliability, and validity of the PEmb-QoL [6,14,16]. Baseline characteristics were shown as proportions or medians and ranges, as appropriate. The transformed PEmb-QoL dimension scores were depicted in a box plot as medians with interquartile range (IQR).

Because acceptability affects the quality of the data obtained, it was assessed by examining completeness of data and score distribution. To examine floor and ceiling effects, we calculated the proportion of patients who achieved the lowest or highest possible score per dimension and in the overall PEmb-QoL summary score. Criteria for acceptability included <15% floor and ceiling effects for dimensions and summary score [14].

Factor analysis is widely used to evaluate whether questionnaire items can be grouped into clusters representing different dimensions of the construct under study [21]. Because the factor analysis in the validation study by Klok et al. [8] did not explore the appropriate number of latent factors (underlying dimensions) but rather sought to confirm the pre-specified structure of the questionnaire based on six dimensions, we explored the number of underlying dimensions and grouping of items in an exploratory factor analysis. We used the principal factor method to analyze the correlation matrix and applied an orthogonal varimax rotation on the loading matrix. The number of retained factors was determined by a scree test [22], which suggested three latent factors with eigenvalues of 17.0, 3.9, and 1.7. After rotation, the three factors accounted for 35%, 22%, and 19% of the total variance, respectively. Question 4a (‘Do your lung symptoms now limit you in daily activities at work?’) was omitted from the factor analysis because almost half of patients in our sample did not work. An item was considered to load on a given factor if the loading was >0.3 for this factor.

We assessed reliability by determining internal consistency, which was measured by Cronbach’s alpha, average inter-item correlation, item-total correlation, and the association between dimensions of the PEmb-QoL scores using pairwise Spearman correlation coefficients. Internal consistency refers to the extent to which items comprising the score measure the same construct (i.e., homogeneity of the score), and was considered acceptable when Cronbach’s alpha was between 0.7 and 0.95 [14]. We regarded an item-total correlation >0.2 and an average inter-item correlation >0.3 as good [23].

We tested reproducibility by repeating the PEmb-QoL questionnaire after ten days (test-retest reliability). Test-retest reliability measures the degree to which repeated measurements applied to the same individuals provide similar answers. We decided that a time period of ten days between the repeated distributions of the questionnaires was long enough to prevent recall bias but short enough to ensure that a clinical change in the symptoms being measured was unlikely to occur. Test-retest reliability was expressed as an intra-class correlation coefficient, with values >0.7 indicating good test-retest reliability [14].

Construct validity refers to the extent to which PEmb-QoL scores relate to other measures in a manner consistent with theoretically derived hypotheses [14]. We first assessed construct validity by calculating pairwise Spearman correlation coefficients between PEmb-QoL and SF-36 dimension and summary scores (convergent validity), as done in previous studies [7,8]. Because both questionnaires were developed to assess health-related quality of life, we assumed that the two measures would be correlated in a moderate range, one being a disease-specific and the other a generic health-related quality of life questionnaire.

We assessed discriminant validity by examining whether PEmb-QoL scores were correlated with measures of unrelated constructs, i.e. patient age, sex, and clinical characteristics (obesity, cancer, and cardiopulmonary diseases). A similar approach was used in previous studies examining discriminant validity of a similarly structured quality of life questionnaire for DVT [6,16]. Our hypothesis was that correlation with age, gender, and clinical characteristics would be weak.

## Results

### Patient sample

Of 242 patients with PE screened, 61 could not be reached, 42 refused to participate, 20 had dementia, 8 lived in a nursing home, 6 were unable to speak French, and 3 lived abroad, leaving a final study sample of 102 patients. Overall, 46 patients (45%) were aged ≥65 years, and 39% were women (Table 1). The median (range) time between occurrence of the index PE and study enrollment was 15 (5–23) months.

**Table 1:** **Baseline patient characteristics (n = 102)**

**Characteristic**	**n (%) or median (range)**
Age, years	63 (26–93)
Female gender	40 (39)
Obesity*	13 (13)
Active cancer	10 (10)
Cardiopulmonary comorbidity^†^	13 (13)
History of venous thromboembolism	18 (18)
Central pulmonary embolism	10 (10)
Time since pulmonary embolism (months)	15 (5–23)

*Body mass index >30 kg/m^2^.

^†^Any cardiac disease with systolic or diastolic ventricular dysfunction or any obstructive or restrictive pulmonary disease.

### Psychometric characteristics of the French version of the PEmb-QoL questionnaire

#### Acceptability

All questionnaires were returned by participants (response rate 100%). We contacted 33 participants (32%) by phone to complete missing items. Overall, 41 participants (40%) answered ‘I do not work’ for the question Q4a.

**Figure 1:**
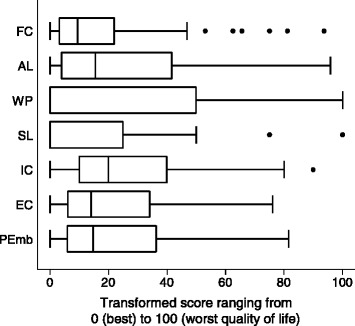
**Box plot of PEmb-QoL dimension and summary scores.** Median transformed scores were 9.4 (interquartile range [IQR] 3.1-21.9) for frequency of complaints (FC), 15.4 (IQR 3.8-41.7) for activities of daily living limitations (AL), 0.0 (IQR 0.0-50.0) for work-related problems (WP), 0.0 (IQR 0.0-25.0) for social limitations (SL), 20.0 (IQR 10.0-40.0) for intensity of complaints (IC), 14.0 (IQR 6.0-34.0) for emotional complaints (EC), and 14.7 (IQR 5.9-36.3) for the PEmb-QoL summary score (PEmb).

The median PEmb-QoL dimension and overall summary scores are shown in Figure 1. All dimensions had floor effects, ranging from 13% for emotional complaints (Q9) to 66% for social limitations (Q6) (Table 2). Ceiling effects were ≤2% for all dimensions except for work-related problems (18%).

**Table 2:** **Floor and ceiling effects of the PEmb-QoL dimension and summary scores**

	Floor effect*	**Ceiling effect** ^**†**^
	**Percent**	
Frequency of complaints (Q 1)	25	0
Activities of daily living limitations (Q 4)	20	0
Work-related problems (Q 5)	60	18
Social limitations (Q 6)	66	2
Intensity of complaints (Q 7 and Q 8)	20	0
Emotional complaints (Q 9)	13	0
PEmb-QoL summary score	3	0

*Indicates the percentage of patients with the lowest possible score, i.e., with the best quality of life.

^†^Indicates the percentage of patients with the highest possible score, i.e., with the lowest quality of life.

#### Factor analysis

We explored the number of underlying dimensions and grouping of items in an exploratory factor analysis, which suggested three latent factors (dimensions): limitations in daily activity (items 4b-m, 5a-d), symptoms (items 1a-h and 7), and emotional complaints (items 9a-f and j) (Table 3). Three items were not clearly assignable to a meaningful dimension in the French version of the PEmb-QoL: item 6 (“During the past four weeks, to what extend have your lung symptoms interfered with your normal social activities with family, friends, neighbors, or groups?”), item 8 (“How much breathlessness have you experienced in the past four weeks?”) and item 9 g-i (“How much of the time during the past four weeks (g) did you feel that you were a burden to your family and friends, (h) were you afraid to exert yourself, (i) did you feel limited in taking a trip?”).

**Table 3:** Factor analysis of the PEmb-QoL questionnaire*

		**Factor 1**	**Factor 2**	**Factor 3**
**Item**			**Factor loadings** ^**†**^	
1a	Pain behind or between the shoulder blades?	0.1031	**0.6572**	0.0632
1b	Pain on or in the chest?	0.0770	**0.7807**	0.2031
1c	Pain in the back?	0.2416	**0.6543**	0.1467
1d	Sensation of pressure?	0.2262	**0.6425**	0.2495
1e	Feeling that there is “still something there”?	0.2990	**0.6354**	0.2826
1f	“Burning sensation” in the lung?	0.09300	**0.7339**	0.2218
1 g	“Nagging feeling”?	0.1382	**0.7831**	0.2318
1 h	Difficulty in breathing or breathlessness?	0.4637	**0.5088**	0.3665
4b	Daily activities at home	**0.7796**	0.3230	0.2529
4c	Social activities	**0.7123**	0.1880	0.2811
4d	Vigorous activities	**0.7471**	0.2588	0.1131
4e	Moderate activities	**0.8412**	0.1479	0.1001
4f	Lifting or carrying activities	**0.8319**	0.1576	0.1946
4 g	Climbing several flights of stairs	**0.7851**	0.2177	0.2789
4 h	Climbing one flight of stair	**0.8031**	0.0575	0.2114
4i	Bending, kneeling, or squatting	**0.6167**	0.1355	−0.0389
4j	Walking more than half a mile	**0.8121**	0.1190	0.0929
4 k	Walking a couple of hundred yards	**0.8235**	−0.0700	0.0380
4 l	Walking about one hundred yards	**0.6313**	−0.0149	0.0573
4 m	Washing or dressing yourself	**0.6052**	−0.0302	0.1083
5a	Cut down the amount of time you spent on work or other activities	**0.5441**	0.2653	0.3327
5b	Accomplished less than you would like	**0.5570**	0.3432	0.3364
5c	Were limited in the kind of work or other activities	**0.5898**	0.3545	0.3838
5d	Had difficulty performing the work or other activities	**0.5865**	0.3976	0.3608
6	Interference with normal social activities	0.4729	**0.5088**	0.4595
7	Intensity of pain around shoulder blades or in chest	0.1619	**0.7540**	0.3329
8	Intensity of breathlessness	**0.5201**	0.3874	0.5030
9a	Worried about having another pulmonary embolism?	−0.0201	0.2722	**0.7017**
9b	Felt irritable?	0.2748	0.3324	**0.7112**
9c	Worried if having to stop anticoagulant medication?	−0.0401	0.1433	**0.5288**
9d	Became emotional more readily?	0.2299	0.1613	**0.8114**
9e	Bothered becoming emotional more readily?	0.2305	0.2645	**0.7739**
9f	Were depressed or in low spirits?	0.3078	0.4111	**0.5902**
9 g	Felt being a burden to family and friends?	**0.5021**	0.3905	0.4279
9 h	Were afraid to exert yourself?	**0.5391**	0.4042	0.4742
9i	Felt limited in taking a trip?	**0.6300**	0.3516	0.4126
9j	Were afraid of being alone?	0.3926	0.1675	**0.6115**

*The scree test suggests three latent factors with eigenvalues of 17.0, 3.9, and 1.7, respectively. After rotation, the three factors accounted for 35%, 22%, and 19% of total variance.

^†^Numbers in bold indicate the highest factor loadings (>0.3) for each item.

#### Reliability and reproducibility

Almost all Cronbach’s alpha coefficients were >0.9 except one (intensity of complaints, 0.7), indicating high internal consistency (Table 4). Items were positively correlated with each other, with all average inter-item correlations >0.3. All item-total correlation values were >0.2, ranging from 0.53 to 0.92 (data not shown for individual items). PEmb-QoL dimensions were moderately well correlated between themselves (0.53 ≤ r ≤0.83), with the highest correlation being between intensity of complaints and frequency of complaints (r = 0.83) and between intensity of complaints and emotional complaints (r = 0.75). Intra-class correlation coefficients for the test-retest analysis were high, ranging between 0.85 for social limitations and 0.96 for emotional complaints (Table 5).

**Table 4:** **Internal consistency reliability**

	**PEmb-QoL questions**	**Number of items**	**Cronbach’s alpha**	**Average inter-item correlation (r)**
**Frequency of complaints**	Q1	8	0.90	0.52
**Activities of daily living limitations**	Q4	13	0.95	0.60
**Work-related problems**	Q5	4	0.91	0.72
**Social limitations**	Q6	1	-	-
**Intensity of complaints**	Q7, Q8	2	0.70	0.54
**Emotional complaints**	Q9	10	0.92	0.52
**PEmb-QoL summary score**	Q1, Q4, Q5, Q6, Q7, Q8, Q9	38	0.92	0.44

**Table 5:** **Test-retest reliability**

	**Intra-class correlation coefficient (95% confidence interval)**
Frequency of complaints	0.94 (0.92-0.96)
Activities of daily living limitations	0.91 (0.87-0.94)
Work-related problems	0.88 (0.84-0.93)
Social limitations	0.85 (0.79-0.90)
Intensity of complaints	0.93 (0.90-0.95)
Emotional complaints	0.96 (0.94-0.97)
PEmb-QoL summary score	0.96 (0.95-0.98)

#### Construct validity (convergent, discriminant)

We did a correlation analysis using SF-36 component scores to assess convergent validity of the PEmb-QoL dimension and overall summary scores. The PEmb-QoL dimensions activities of daily living limitations, work-related problems, social limitations, and intensity of complaints showed higher correlations with the SF-36 Physical Component Summary, whereas frequency of complaints and emotional complaints had higher correlations with the SF-36 Mental Component Summary (Table 6). Overall, these correlations supported a good convergent validity. The PEmb-QoL dimension and overall summary scores were only weakly correlated with clinical characteristics, indicating a good discriminant validity (Table 7).

**Table 6:** Pairwise spearman correlations between SF-36 and PEmb-QoL dimension/summary score*

**PEmb-QoL**	**SF-36 physical functioning**	**Physical role functioning**	**Bodily pain**	**General health**	**Vitality**	**Social functioning**	**Emotional role functioning**	**Mental health**	**Physical health summary**	**Mental health summary**
**Frequency of complaints**	0.47	0.51	0.68	0.47	0.60	0.56	0.49	0.61	0.49	0.59
**ADL limitations**	0.90	0.72	0.61	0.57	0.54	0.56	0.64	0.40	0.80	0.39
**Work-related problems**	0.62	0.80	0.66	0.47	0.58	0.63	0.59	0.42	0.70	0.46
**Social limitations**	0.62	0.70	0.61	0.54	0.57	0.69	0.58	0.53	0.64	0.56
**Intensity of complaints**	0.59	0.58	0.72	0.50	0.66	0.67	0.51	0.56	0.60	0.58
**Emotional complaints**	0.53	0.68	0.65	0.55	0.62	0.83	0.71	0.71	0.54	0.76
**PEmb-QoL summary score**	0.80	0.77	0.75	0.61	0.69	0.75	0.68	0.60	0.76	0.61

Abbreviations: *ADL* = Activities of daily living.

*Numbers represent Spearman correlation coefficients (r). The SF-36 summary and dimension scores were reversed for this analysis, i.e. the lower the score, the better the quality of life. All correlations between dimension/summary scores were statistically significant (*P* <0.001).

**Table 7:** **Pairwise spearman correlations between PEmb-QoL dimensions/summary score and baseline characteristics** *

	**Age**	**Female gender**	**Obesity**	**Cancer**	**Cardiopulmonary comorbidity** ^**‡**^
**Frequency of complaints**	−0.05	0.07	0.21†	−0.15	0.03
**ADL limitations**	0.32†	−0.08	0.18	−0.07	0.12
**Work-related problems**	0.18	−0.16	−0.02	−0.05	0.17
**Social limitations**	0.02	0.01	0.21†	−0.12	0.11
**Intensity of complaints**	−0.01	−0.06	0.14	−0.16	0.10
**Emotional complaints**	−0.10	−0.07	0.13	−0.18	0.13
**Overall PEmb-QoL summary score**	0.12	−0.07	0.16	−0.15	0.12

Abbreviations: *ADL* = Activities of daily living.

*Numbers represent Spearman correlation coefficients (r).

^†^Correlation with statistical significance (*P* <0.05).

^‡^Any cardiac disease with systolic or diastolic ventricular dysfunction or any obstructive or restrictive pulmonary disease.

## Discussion

In our validation study, the PEmb-QoL questionnaire showed not only a high internal consistency and inter-item and item-total correlation but also high test-retest reliability. The high score correlations between the PEmb-QoL and the SF-36 and the low correlation between PEmb-QoL scores and patient characteristics supported convergent and discriminant validity, respectively. Thus, the French version of the PEmb-QoL questionnaire met standard criteria of reliability and validity for use as a patient-reported measure of outcome in patients with PE, as previously shown for the Dutch version of the questionnaire.

Only one dimension, work related problems, had a substantial ceiling effect, with 18% of patients scoring the maximum score (lowest possible quality of life) in this dimension. In contrast, a substantial floor effect was present in five out of six PEmb-QoL dimensions, i.e. more than 15% of patients had the lowest score possible, indicating the best possible quality of life. We could not exclude the possibility that the Likert scale used did not have a large enough range to accommodate the distribution of the data or that there was a social desirability bias (i.e., patients thought it made them look better if they reported high quality of life). The exceptionally high floor effect (66%) of social limitations could be explained by the fact that this dimension consists of a single question only. Given that Klok et al. [8] already observed such floor and ceiling effects in some of the PEmb-QoL dimensions, we presumed that the floor and ceiling effects were not specifically related to the French version of the PEmb-QoL questionnaire. Indeed, floor effects >15% were also observed in all six dimensions in the Norwegian version of the PEmb-QoL [10].

When correlating the PEmb-QoL and the SF-36 dimensions, we found a particularly strong correlation between activities of daily living limitations and physical functioning, and between work-related problems and physical role functioning. A similar observation had been made by Klok et al. [8] and might be explained by the fact that these dimensions focus on the extent of limitations when performing work or physical exercises. The relatively strong correlation between emotional complaints and social and emotional role functioning and mental health did not come as a surprise, either. The correlation between intensity of complaints and bodily pain was clinically also plausible.

The six dimensions of the PEmb-QoL were originally defined clinically and not statistically, assuming that these six dimensions would provide unique information to the treating physician [7]. The factor analysis presented by Klok et al. [8] showed that items designated to social limitations and intensity of complaints had higher loadings in other dimensions, suggesting that these two dimensions might not be justifiable. Our factor analysis supported the formation of three dimensions: limitations in daily activity (items 4b-m, 5a-d), symptoms (items 1a-h and 7), and emotional complaints (items 9a-f and j). Notably, items 6 (Interference with normal social activities), 8 (Intensity of breathlessness), and 9 g-i (Burden to family and friends, Afraid to exert yourself, Limited in taking a trip) were not taken into account in these three dimensions because these did not contribute significantly to either of them. Given that items 6, 8, 9 g, and 9i did not have the highest loadings in the original version of the PEmb-QoL either, a possibility would be to remove items 6, 8, 9 g, and 9i and to replace the original six dimensions by three dimensions in the French version of the questionnaire. As an alternative, items 6, 8, 9 g-i could be grouped into the dimension on which they loaded highest, i.e. item 6 (Interference with normal social activities) in symptoms and items 8 (Intensity of breathlessness) and 9 g-i (Burden to family and friends, Afraid to exert yourself, Limited in taking a trip) in limitations in daily activity. Either way, further validations of this adapted PEmb-QoL questionnaire would be necessary. In the Norwegian version of PEmb-QoL questionnaire, an exploratory factor analysis using a different criterion to determine the number of underlying dimensions (Eigenvalues >1) resulted in six new dimensions that were not identical with the original version [10]. It is well known that measurement properties of QoL questionnaires adapted for a different population may differ from their original version due to differing translational, cultural, and methodological factors [11,21,24]. While items 9 g-i (Burden to family and friends, Afraid to exert yourself, Limited in taking a trip) were expected to cluster in the dimension “emotional complaints”, they clustered in “limitations in daily activity” in our study. A potential explanation is that the wordings “burden”, “exert yourself”, and “taking a trip” are not interpreted as emotional issues but rather as an obstacle to daily activity. Given that patients in our cohort were older (63 vs. 56 years) and less likely to have cardiopulmonary comorbidity (13% vs. 20%) and obesity (13% vs. 39%) than patients in the study by Klok et al. [8], we could not exclude the possibility that these differences in patient baseline characteristics did not contribute to differences in self-reported health measures.

To facilitate the comparison of PE-related quality of life across studies, we created an overall PEmb-QoL summary score using all items, except Q2 (“At what time of day are your lung symptoms most intense?”) and Q3 (“Compared to one year ago, how would you rate the condition of your lungs in general now?”), which were never scored. This overall score met standard criteria of acceptability, reliability, reproducibility, and validity for use as a summary patient-reported measure of outcome in patients with acute PE.

Our study has potential limitations. First, only 42% of potentially eligible persons with PE completed the questionnaires, mostly, because they were unreachable or refused to participate. Thus, we could not entirely exclude the possibility that elderly and sicker patients were underrepresented in our study. However, our enrolment rate compared well with previous studies, in which less than 40% of screened patients with VTE underwent quality of life assessments [10,25]. Second, we could not ascertain the number of missing items per patient because missing items were completed by participants following contact by phone with a study collaborator. However, less than a third of patients had to be contacted because they had one or more missing items. Finally, we were not able to assess responsiveness of the PEmb-QoL questionnaire, that is, its ability to detect a clinically meaningful change over time [8].

In conclusion, despite the presence of some floor and ceiling effects, the French version of the PEmb-QoL questionnaire meets standard criteria of reliability and validity for use as a patient-reported measure of quality of life and symptoms in patients with PE. Thus, the French version of the PEmb-QoL can be used with confidence in prospective studies to assess PE-specific quality of life and symptoms.

## Additional file

Additional file 1:
**The French version of the PEmb-QoL questionnaire.**
