# Supplementary material for: Quality of life after pulmonary embolism: validation of the French version of the PEmb-QoL questionnaire
Source: Health Qual Life Outcomes. 2014 Dec 3;12:174. doi: 10.1186/s12955-014-0174-4 (PMC4260180; doi:10.1186/s12955-014-0174-4)
Supplement: Additional file 1: — The French version of the PEmb-QoL questionnaire. [file 12955_2014_174_MOESM1_ESM.pdf]

PEmb-QoL
QUESTIONNAIRE

Questionnaire

après avoir eu une

EMBOLIE PULMONAIRE

INSTRUCTIONS POUR REPONDRE:
Veuillez répondre à toutes les questions. Si vous ne savez pas comment répondre, choisissez la réponse la plus proche de votre situation.

Ce questionnaire concerne **vos poumons**. Les informations que vous allez donner doivent décrire ce que vous ressentez. Vous pouvez aussi indiquer si vous vous sentez capable de réaliser des activités quotidiennes normales.

1.	Au cours de ces <u>4 dernières semaines</u>, combien de fois avez-vous ressenti les plaintes pulmonaires suivantes?					
	(cocher la réponse devotre choix, une par ligne)	**Tous les jours**	**Plusieurs fois par semaine**	**Environ une fois par semaine**	**Moins d'une fois par semaine**	**Jamais**
1.a	Douleur(s) derrière ou entre les omoplates ?	☐1	☐2	☐3	☐4	☐5
1.b	Douleur(s) dans ou sur la poitrine ?	☐1	☐2	☐3	☐4	☐5
1.c	Douleur(s) dans le dos ?	☐1	☐2	☐3	☐4	☐5
1.d	Sensation d'oppression thoracique?	☐1	☐2	☐3	☐4	☐5
1.e	Sensation qu'il y a toujours « quelque chose là» ?	☐1	☐2	☐3	☐4	☐5
1.f	Sensation de brulûres aux poumons ?	☐1	☐2	☐3	☐4	☐5
1.g	Sensation lancinante aux poumons ?	☐1	☐2	☐3	☐4	☐5
1.h	Difficulté à respirer ou essouflement ?	☐1	☐2	☐3	☐4	☐5

2.	A quel moment de la journée vos **plaintes pulmonaires** sont-elles **les plus intenses**? *(cocher la réponse de votre choix)*		
☐1	Au réveil	☐4	Pendant la nuit
☐2	A la mi-journée	☐5	A tout moment de la journée
☐3	En fin de journée	☐6	Jamais

3.	<u>Par rapport à l'année dernière à la même époque</u>, comment évaluez-vous l'état de **vos poumons** <u>en ce moment</u>? *(cocher la réponse de votre choix)*		
☐1	Bien meilleurs maintenant que l'an dernier	☐4	Plutôt moins bon maintenant que l'an dernier
☐2	Plutôt meilleurs maintenant que l'an dernier	☐5	Beaucoup moins bon maintenant que l'an dernier
☐3	A peu près pareils maintenant que l'an dernier	☐6	Je n'avais aucun problème pulmonaire

4.	Voici une liste d'activités que vous pouvez avoir à faire dans votre vie de tous les jours.<br>Est-ce que **<u>vos plaintes pulmonaires</u>** <u>limitent</u> les activités suivantes ?<br>Si tel est le cas, combien êtes-vous limité ?				
	(cocher la réponse de votre choix, une par ligne)	**Je ne travaille pas**	**OUI, très limité**	**OUI, un peu lmité**	**NON, pas du tout limité**
4.a	Activités quotidiennes au travail	☐0	☐1	☐2	☐3
4.b	**Activités quotidiennes domestiques**<br>(par exemple: faire le ménage, repasser, bricoler, jardiner, etc...)		☐1	☐2	☐3
4.c	**Activités sociales ou de loisirs** (par exemple: voyager, aller au cinéma, aller à une soirée, faire des courses, etc...)		☐1	☐2	☐3
4.d	**Effort physique important** (par exemple : courir, soulever des charges lourdes, faire une activité sportive intense)		☐1	☐2	☐3
4.e	**Effort physique modéré** (par ex. déplacer une table, passer l'aspirateur, nager ou faire du vélo)		☐1	☐2	☐3

4.f	Monter ou porter les courses	☐1	☐2	☐3
4.g	Monter **plusieurs** étages d'escaliers	☐1	☐2	☐3
4.h	Monter **un** étage d'escaliers	☐1	☐2	☐3
4.i	Vous pencher en avant, vous agenouiller, vous accroupir	☐1	☐2	☐3
4.j	Marcher **plus de 800 mètres**	☐1	☐2	☐3
4.k	Marcher **quelques centaines de mètres**	☐1	☐2	☐3
4.l	Marcher **environ 100 mètres**	☐1	☐2	☐3
4.m	Vous laver et vous habiller seul(e)	☐1	☐2	☐3

**5.**	Au cours de ces 4 dernières semaines, avez-vous eu les problèmes suivants dans votre travail ou dans vos activités de tous les jours en raison de **vos plaintes pulmonaires**?		
	(cocher la réponse de votre choix, une par ligne)	**OUI**	**NON**
5.a	Avez-vous réduit **le temps** passé à votre travail ou à vos activités habituelles?	☐1	☐2
5.b	Avez-vous **fait moins de choses** que ce que vous auriez souhaité?	☐1	☐2
5.c	Avez-vous été limité pour faire **certaines** choses à votre travail ou lors de vos activités habituelles?	☐1	☐2
5.d	Avez-vous eu des **difficultés** à faire votre travail ou tout autre activité (par exemple cela vous a-t-il demandé un effort supplémentaire)?	☐1	☐2

**6.**	Au cours de ces 4 dernières semaines, dans quelle mesure vos **plaintes pulmonaires** vous ont-elles gêné dans vos activités sociales habituelles avec votre famille, vos amis, vos voisins ou d'autres personnes ?		
	(cocher la réponse de votre choix)	☐3	Moyennement
☐1	Pas du tout	☐4	Beaucoup
☐2	Un petit peu	☐5	Enormément

**7.**	Quelle a été l'importance de vos douleurs dans la région des omoplates/ dans la poitrine, au cours de ces 4 dernières semaines? *(cocher la réponse de votre choix)*		
☐1	Nulle	☐4	Moyenne
☐2	Très faible	☐5	Grande
☐3	Faible	☐6	Très grande

**8.**	Au cours de ces 4 dernières semaines, quelle a été l'importance de votre essouflement? *(cocher la réponse de votre choix)*		
☐1	Nulle	☐4	Moyenne
☐2	Très faible	☐5	Grande
☐3	Faible	☐6	Très grande

9.	Les questions qui suivent portent sur comment vous vous êtes senti(e) au cours de ces 4 dernières semaines en raison de vos **plaintes pulmonaires**. Pour chaque question, merci d'indiquer la réponse qui vous semble la plus appropriée.<br>Au cours de ces 4 dernières semaines, y a-t-il eu des moments où:						
	(cocher la réponse de votre choix, une par ligne)	**En permanence**	**Très souvent**	**Souvent**	**Quelquefois**	**Rarement**	**Jamais**
a.	Vous avez été préoccupé(é) par l'idée d'avoir une nouvelle embolie pulmonaire ?	☐1	☐2	☐3	☐4	☐5	☐6
b.	Vous vous êtes senti(e) irritable?	☐1	☐2	☐3	☐4	☐5	☐6
c.	Vous seriez inquiet(e) à l'idée de devoir arrêter vos médicaments anticoagulants?	☐1	☐2	☐3	☐4	☐5	☐6
d.	Vous êtes devenu(e) plus facilement émotif/émotive ?	☐1	☐2	☐3	☐4	☐5	☐6
e.	Est-ce que cela vous a dérangé d'être plus rapidement émotif/émotive ?	☐1	☐2	☐3	☐4	☐5	☐6
f.	Vous avez été déprimé(e) ou avec un moral bas ?	☐1	☐2	☐3	☐4	☐5	☐6
g.	Vous vous êtes senti(e) comme un fardeau pour votre famille ou vos amis?	☐1	☐2	☐3	☐4	☐5	☐6
h.	Vous avez eu peur peur de faire des efforts physiques ?	☐1	☐2	☐3	☐4	☐5	☐6
i.	Vous vous êtes senti(e) limité(e) pour faire une excursion ?	☐1	☐2	☐3	☐4	☐5	☐6
j.	Vous avez eu peur d'être seul(e) ?	☐1	☐2	☐3	☐4	☐5	☐6

Nous vous remercions pour votre collaboration.
